# Description of the First Case of Adenomyomatosis of the Gallbladder in an Infant

**DOI:** 10.1155/2014/248369

**Published:** 2014-06-15

**Authors:** Yuri A. Zarate, Katherine A. Bosanko, Chaowapong Jarasvaraparn, Jaime Vengoechea, Elizabeth M. McDonough

**Affiliations:** ^1^Section of Genetics and Metabolism, University of Arkansas for Medical Sciences College of Medicine, Little Rock, AR 72205, USA; ^2^Arkansas Children's Hospital, 1 Children's Way, Slot 512-22, Little Rock, AR 72202, USA; ^3^Division of Pediatric Gastroenterology, Hepatology and Nutrition, University of Arkansas for Medical Sciences College of Medicine, Little Rock, AR 72205, USA

## Abstract

We report here the case of the youngest patient with adenomyomatosis of the gallbladder in a female infant diagnosed at 4 months of age. This diagnosis was made based on characteristic ultrasonography findings in a patient that was undergoing routine surveillance for a suspected clinical diagnosis of Beckwith-Wiedemann syndrome. The patient remains asymptomatic and currently no surgical interventions have been needed. We review the pathophysiology and ultrasonographic findings of this rare condition and present a comparison with the only other four pediatric cases of adenomyomatosis of the gallbladder.

## 1. Introduction 

Adenomyomatosis of the gallbladder (ADMG) is an acquired condition characterized by localized or diffuse epithelial proliferation and invagination of the mucosa through a hypertrophied muscularis, forming intramural diverticula or sinus tracts. ADMG is diagnosed mainly by imaging. The pathogenesis, pathology, and indications for surgery in this condition are not well understood, especially in children. Although ADMG is benign in nature, it is possible that lithiasis and chronic inflammation secondary to ADMG may lead to dysplastic changes and cancer [1].

We report the case of a female infant diagnosed with ADMG at 4 months of age based on characteristic ultrasonography findings. This diagnosis was made incidentally on a patient that was undergoing routine surveillance for Beckwith-Wiedemann syndrome (BWS). This represents the first report of concurrent BWS and ADMG. Moreover, our case is an infant and to our knowledge is the youngest patient reported with ADMG.

## 2. Patient Presentation

The female proband was the first child of nonconsanguineous biracial parents (Caucasian and Brazilian). As a product of a naturally conceived full term pregnancy, she was born by cesarean section and weighed 4.932 kg (>97th centile) with no prenatal or perinatal complications. She was first evaluated at 2 months of age for possible diagnosis of BWS given her macrosomia and positive family history. Her weight was 7.2 kg (>97th centile), length was 61 cm (93rd centile), and head circumference was 42.2 cm (98th centile). There was no history of omphalocele or neonatal hypoglycemia and, on physical exam, no hemihyperplasia, ear lobe creases, or macroglossia. Dysmorphic features included narrow palpebral fissures, epicanthal folds, and tented vermilion of the upper lip.

She was subsequently evaluated at 7 months of age with persistent macrosomia. She is otherwise healthy and with normal developmental history. To complete the genetic workup, we also performed a whole genome array (Cytoscan HD, Affymetrix) that was normal. The infant received the clinical diagnosis of BWS and was started on the recommended surveillance protocol. Her alpha-fetoprotein levels (AFP) were 71.2 IU/mL and 22.6 IU/mL at five and eight months of age, respectively (Normal 0–7.2 IU/mL). Liver enzymes and renal ultrasounds have been normal.

**Figure 1:**
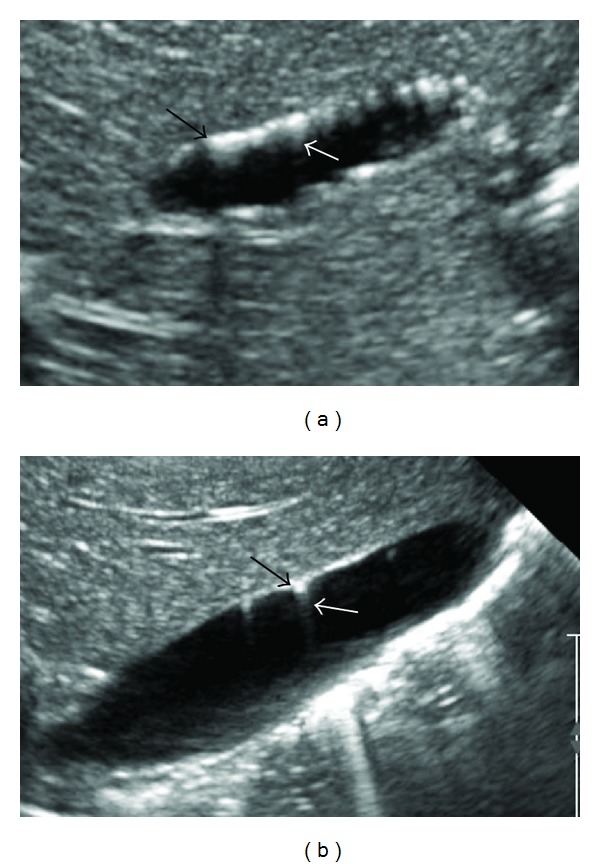
Ultrasound images at 4 months of age (a) and 7 months of age (b). Multiple nondependent echogenic foci were seen in wall of gall bladder or Rokitansky-Aschoff sinuses (black arrow) with distal sonographic shadowing or comet tail artifacts (gray arrow).

Of particular interest, however, was her initial abdominal ultrasound at 4 months of age (Figure 1(a)). The gallbladder wall was noted to have unusual echogenic foci interpreted as ADMG. A repeat ultrasound at 7 months of age showed the same finding with no change (Figure 1(b)). Since, she has been evaluated by gastroenterology and surgery. Given the lack of clinical signs of complications, the plan is to not intervene and to follow up her gallbladder on routine ultrasounds every 3 months as indicated in BWS.

Her 24-year-old mother presented to genetics clinic 34 weeks into the pregnancy. Given the history of macroglossia, hemihyperplasia, and Wilms tumor, we made a clinical diagnosis of BWS and ordered methylation analysis from peripheral blood (Methylation Specific-Multiplex Ligation Dependent Probe Amplification, MS-MLPA) which was normal. We then ordered* CDKN1C* sequencing which was also normal.

## 3. Discussion 

Hyperplastic cholecystosis is a term used to differentiate from inflammatory conditions such as acute cholecystitis, since it lacks inflammatory features but exhibits features of hyperplasia. It includes two types of mucosal abnormalities of the gallbladder which are usually clinically incidental findings at the time of cholecystectomy: cholesterolosis and adenomyomatosis. Cholesterolosis is characterized by mucosal villous hyperplasia with excessive accumulation of cholesterol esters within epithelial macrophages, while ADMG describes an acquired, hyperplastic lesion of the gallbladder characterized by overgrowth of the mucosa, thickening of the muscle wall, and intramural diverticula [2].

Rokitansky-Aschoff sinuses (RAS) can appear on ultrasound as echo-poor intramural cystic structures. If they contain sludge, they become echogenic. RASs can also produce distal sonographic shadowing or ring-down (comet tail) artifacts. Such ring-down has been proposed to result from reverberation between the near and far surfaces of the sinuses themselves, closely adjacent intrasinus papillary projections, or contained cholesterol crystals. Sonographic findings of ring-down and polypoid projections of <10 mm suggest ADMG [3–5].

Although gallbladder diseases in children are reported with increasing prevalence because of the widespread use of ultrasonography as a diagnostic tool, ADMG in children was first reported in the 1990s and only in four cases to date (Table 1) [1, 6–8]. All four patients were healthy with no underlying disease except for abdominal pain. Moreover, their age range was 5–9 years (3 males and 1 female). To the best of our knowledge, we present the first case of ADMG in an infant, who was evaluated for BWS given the history of macrosomia and a positive family history (two major criteria).

ADMG is currently divided into three types: diffuse, localized, and segmental (annular type). In the localized type (fundal type), a cystic structure forms a nodule, usually in the fundus, that projects into the lumen showing a polyp on ultrasonography [4, 9]. Our patient appeared to have this type. Ultrasonography is a very sensitive and specific image modality but further imaging may be used to inform a surgical decision in cases without infection or cholelithiasis [6, 7]. Given the lack of objective evidence of complications related to ADMG, our patient has not needed further invasive imaging and continues to be monitored clinically.

Although ADMG is benign in nature, stones and chronic inflammation secondary to ADMG may lead to dysplastic changes and cancer, but the causal relationship remains questioned [1, 10]. Surgical intervention for patients with asymptomatic ADMG remains controversial [10]. All four pediatric cases previously reported had undergone cholecystectomy because they experienced abdominal pain. Our child is asymptomatic and requires regular ultrasonographic gallbladder surveillance every 3 months due to her underlying clinical diagnosis of BWS. Therefore, we will monitor her for symptoms and for signs of gallbladder thickening and irregularity, which could be indications for cholecystectomy.

We present the case of the youngest patient with ultrasonographic findings highly suggestive of ADMG with the context of possible BWS. Ultimately, we need better knowledge to determine the long term consequences of ADMG and its potential relationship with gallbladder cancer.

## Figures and Tables

**Table 1:** Previous cases of adenomyomatosis of the gallbladder in children.

Study	Origin	Age (years)	Sex	Symptom	Ultrasonography	Management	Type
(1) Alberti et al., 1998 [6].	Italy	5	Male	Abdominal pain	Echogenic nodule was detected next to the neck.	Laparoscopic cholecystectomy	Localized

(2) Cetinkursun et al., 2003 [7].	Turkey	6	Male	Abdominal pain, fever and bile vomiting.	A small and multiseptated gallbladder with thickened wall.	Open cholecystectomy	Diffuse

(3) Zani et al., 2005 [8].	Italy	5	Male	Abdominal pain	Multiseptated gallbladder within the lumen.	Open cholecystectomy	Segmental (annular type)

(4) Akçam et al., 2008 [1].	Turkey	9	Female	Abdominal pain	Thickening of the wall of the gallbladder with echogenic areas parallel to the wall of gallbladder.	Open cholecystectomy	Diffuse (honeycomb)

(5) Our case, 2014.	USA	4 m	Female	Incidental finding	Echoic foci within gallbladder wall.	Observation	Localized
